# Supplementary material for: Triboiontronics with temporal control of electrical double layer formation
Source: Nat Commun. 2024 Jul 23;15:6182. doi: 10.1038/s41467-024-50518-3 (PMC11263338; doi:10.1038/s41467-024-50518-3)
Supplement: Supplementary file 1 — Supplementary Information [file 41467_2024_50518_MOESM1_ESM.pdf]

Supplementary Information

Triboiontronics with temporal control of electrical double layer formation

Xiang Li [a, b], Roujuan Li [a, b], Shaoxin Li [a], Zhong Lin Wang [a, c, d, e, *], Di Wei [a, f, *]

[a] *Beijing Institute of Nanoenergy and Nanosystems, Chinese Academy of Sciences, Beijing 101400, P. R. China*

[b] *School of Nanoscience and Engineering, University of Chinese Academy of Sciences, Beijing 100049, P. R. China*

[c] *Beijing Key Laboratory of Micro-Nano Energy and Sensor, Center for High-Entropy Energy and Systems, Beijing Institute of Nanoenergy and Nanosystems, Chinese Academy of Sciences, Beijing 101400, P. R. China*

[d] *Guangzhou Institute of Blue Energy, Knowledge City, Huangpu District, Guangzhou 510555, P. R. China*

[e] *Georgia Institute of Technology, Atlanta, GA 30332-0245, United States*

[f] *Centre for Photonic Devices and Sensors, University of Cambridge, 9 JJ Thomson Avenue, Cambridge, CB3 0FA, UK*

*Corresponding authors' e-mail addresses: zhong.wang@mse.gatech.edu, dw344@cam.ac.uk.

This file includes:

Supplementary Figs. 1-38

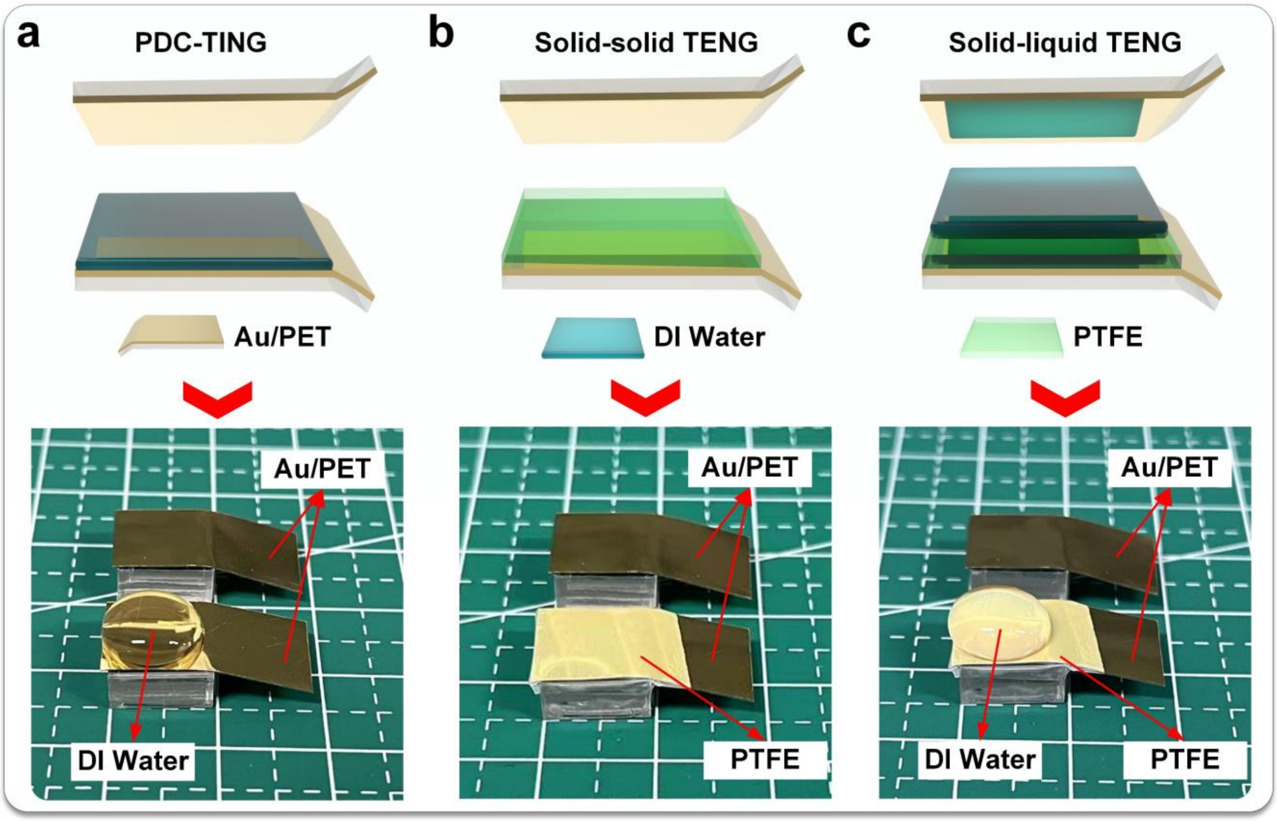

Supplementary Fig. 1 Schematic and physical diagrams of different generators. **a**, When 200 μL deionized (DI) water was dropped on the pristine bottom gold/polyethylene terephthalate (Au/PET) surface, the physically adsorbed direct-current triboiontronic nanogenerator (PDC-TING) was constructed. **b**, When the polytetrafluoroethylene (PTFE) film was used to replace DI water and attached to the bottom Au/PET surface, the conventional solid-solid triboelectric nanogenerator (TENG) was constructed. **c**, When 200 μL DI water was dropped on the PTFE film attached to the bottom Au/PET surface, the conventional solid-liquid TENG was constructed.

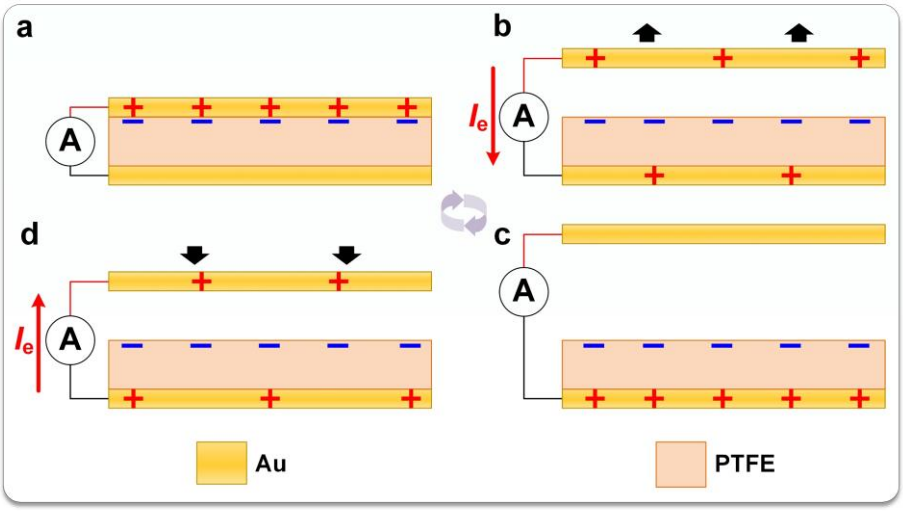

Supplementary Fig. 2 The coupling principle of contact electrification (CE) and electrostatic induction of the solid-sild TENG. **a**, Based on the different electronegativity, when the PTFE dielectric layer first contacted the separate electrode, electrons from the separate Au electrode surface were transferred to the PTFE dielectric layer. **b**, When the separate Au electrode and PTFE dielectric layer gradually separated from each other, electrons on the back Au electrode surface were gradually transferred to the separate Au electrode surface, generating the positive electronic displacement current I_e. **c**, The PTFE dielectric layer was completely separated from the separate Au electrode, and electrons on the back Au electrode surface were completely transferred to the separate Au electrode surface. **d**, When the separate Au electrode was constantly in contact with the PTFE dielectric layer, electrons from the separate Au electrode surface were transferred to the back Au electrode surface, generating the negative electronic displacement current I_e.

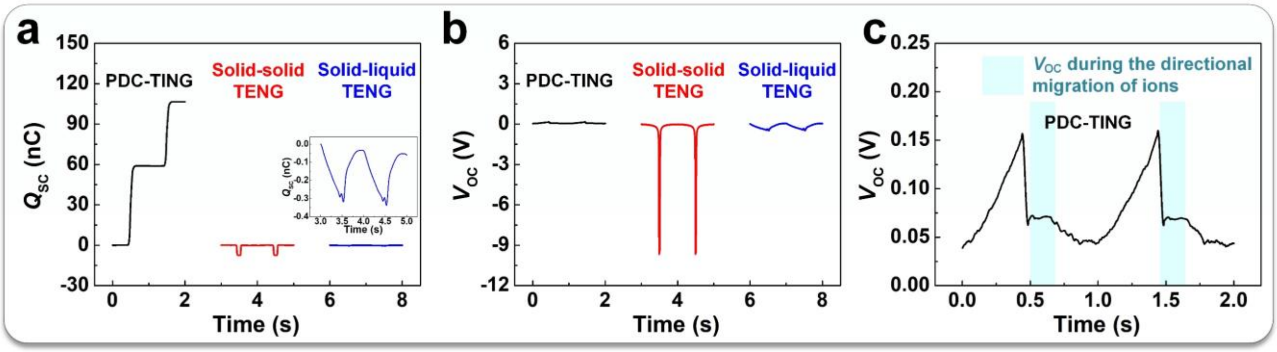

Supplementary Fig. 3 Comparison of output performance of different generators. **a**, The transferred charge (Q_{SC}) generated by PDC-TING, solid-solid TENG, and solid-liquid TENG were 59.00 nC, 7.50 nC, and 0.32 nC, respectively. **b**, The t open-circuit voltage (V_{OC}) generated by PDC-TING, solid-solid TENG, and solid-liquid TENG were 0.073 V, 9.67 V, and 0.48 V, respectively. **c**, The detailed display of the V_{OC} during the directional migration of ions in the PDC-TING. The stages with a blue background represented the V_{OC} generated during the ion migration and the remaining stages represented electronics displacement current signals generated by electrostatic induction. Both the short-circuit current (I_{SC}) and Q_{SC} generated by electrostatic induction were significantly weaker and not clearly depicted in the corresponding signal diagram.

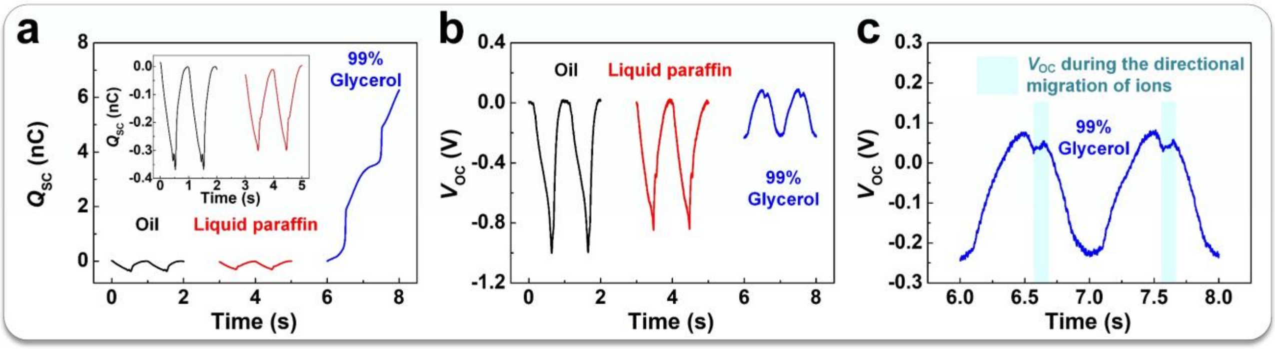

Supplementary Fig. 4 The effect of different liquid types on the PDC-TING output. a, When applying 200 μL of oil or liquid paraffin onto the pristine bottom Au/PET layer, the generated Q_{SC} were 0.37 nC, 0.30 nC, and 3.30 nC, respectively. **b**, The generated V_{OC} were 1.00 V, 0.85 V, and 0.042 V, respectively. **c**, The detailed display of the V_{OC} of 0.042 V during the directional migration of ions in the PDC-TING with 99% glycerol.

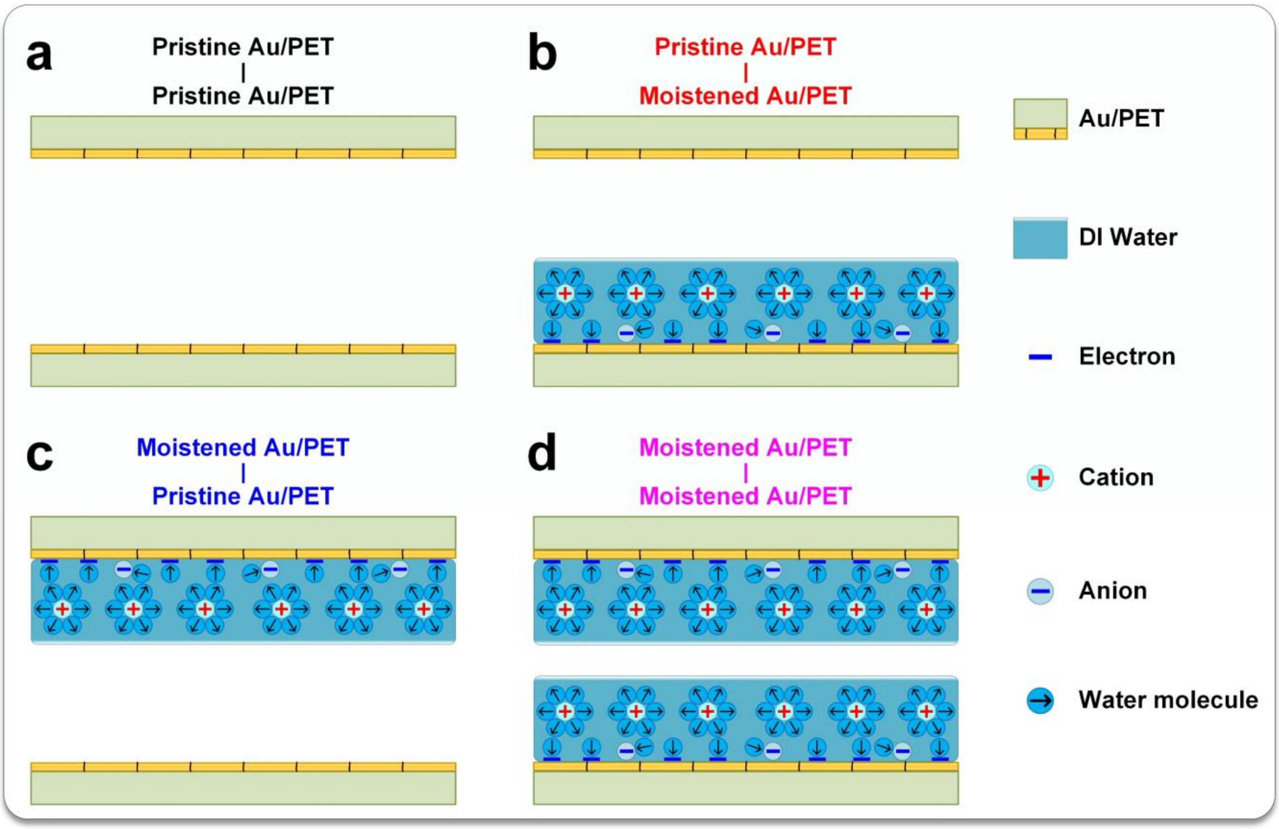

Supplementary Fig. 5 The specific pre-moistening methods of pre-moistening different Au/PET layers. a, The top and bottom Au/PET layers were pristine. **b**, Pre-moistening was exclusively applied to the bottom Au/PET layer with DI water to pre-form a dense EDL. **c**, Pre-moistening was exclusively applied to the top Au/PET layer with DI water to pre-form a dense EDL. **d**, The top and bottom Au/PET layers were pre-moistened.

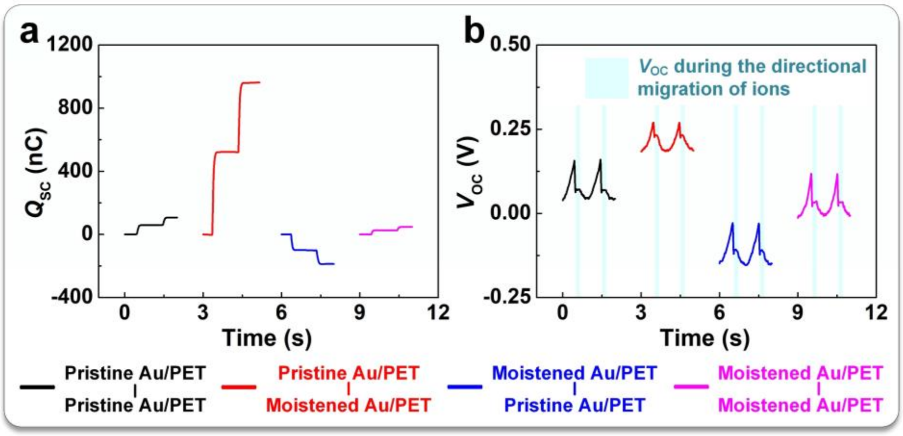

Supplementary Fig. 6 The effect of the extent and direction of asymmetry in the EDL formation on the PDC-TING output was investigated by pre-moistening different Au/PET layers. a, Under the four pre-moistening methods of pristine Au/PET-pristine Au/PET, pristine Au/PET-moistened Au/PET, moistened Au/PET-pristine Au/PET, and moistened Au/PET-moistened Au/PET, the Q_{SC} generated by the PDC-TING were 59.00 nC, 523.15 nC, -100.69 nC, and 26.78 nC, respectively. **b**, Under the four pre-moistening methods, the V_{OC} generated by the PDC-TING were 0.073 V, 0.23 V, -0.11 V, and 0.034 V, respectively.

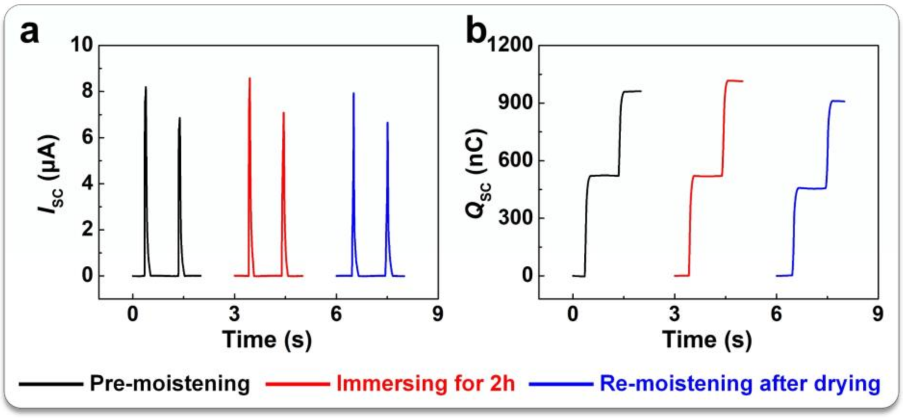

Supplementary Fig. 7 The influence of the swelling property, water content, and dehydration situation of the Au/PET layer on the PDC-TING output was further investigated through systematic experiments. **a**, The I_{SC} of the PDC-TING remained relatively stable at 8.20 μA. **b**, The Q_{SC} remained relatively stable at 523.15 nC. The PDC-TING output might be minimally influenced by the swelling property, water content, and dehydration status of the PET substrate. This observation might be attributed to two primary factors. Firstly, the EDL formation at the interface occurs once the Au/PET layer comes into full contact with DI water, largely independent of the water content within the PET substrate. Secondly, the PET film demonstrates low water absorption, typically ranging from 0.06% to 0.129%, resulting in the relative stability of the Au/PET layer in DI water with minimal swelling or water loss.

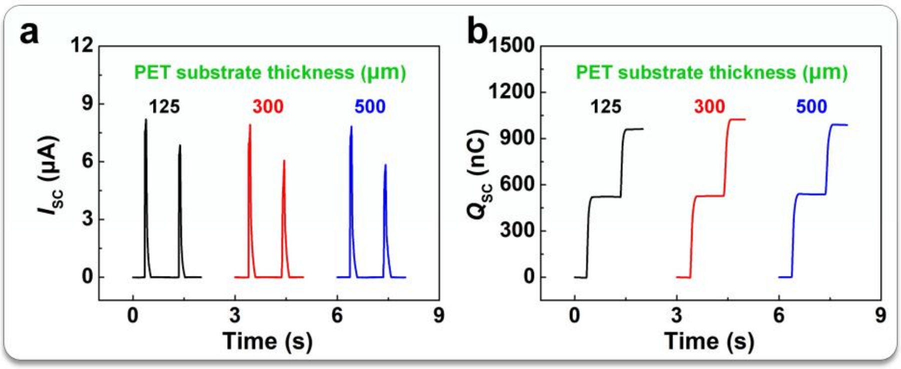

Supplementary Fig. 8 The effect of the thickness of the PET substrate in the Au/PET layer on the PDC-TING output was investigated. a, When keeping the sputtering time of the Au layer for 10 min, increasing the thickness of the PET substrate from 125 μm to 500 μm had minimal effect on the PDC-TING output, and the I_{SC} remained relatively stable with 8.20 μA. **b**, The Q_{SC} remained relatively stable with 523.15 nC.

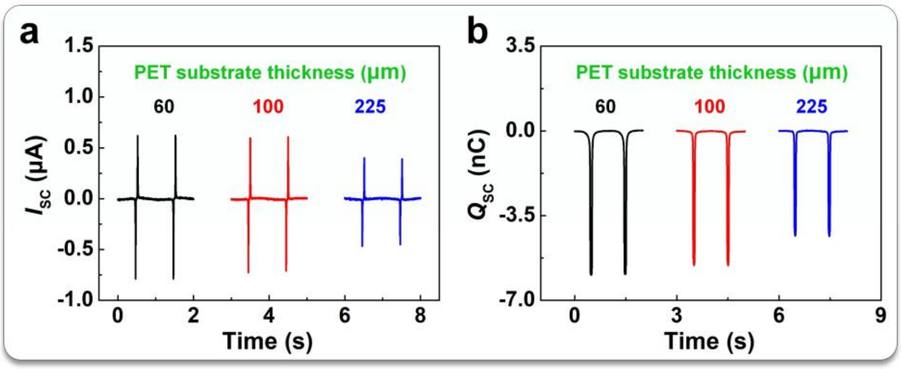

Supplementary Fig. 9 The effect of the thickness of the dielectric material on the conventional solid-solid TENG output was investigated. a, When the dielectric material (PET film) increased from 60 μm to 225 μm, the I_{SC} decreased from about 0.80 μA to 0.47 μA. **b**, The Q_{SC} decreased from about 6.0 nC to 4.3 nC.

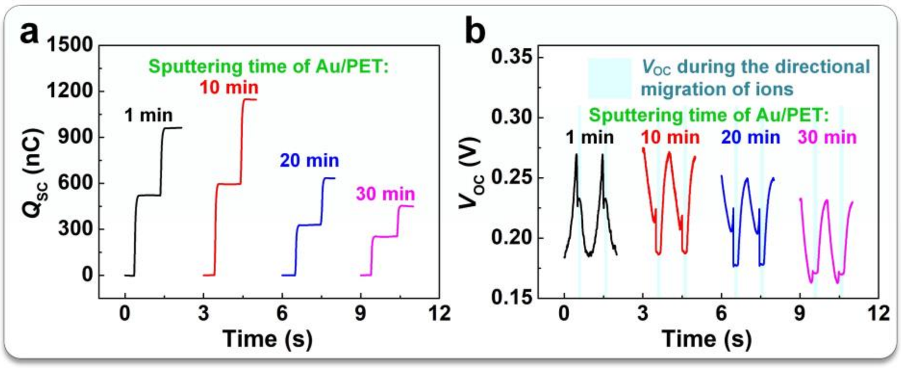

Supplementary Fig. 10 The effect of the Au sputtering time of the Au/PET layer on the PDC-TING output was investigated. a, When the Au sputtering time of the Au/PET layers increased from 1 minute to 10 minutes, the Q_{SC} of the PDC-TING increased from 523.15 nC to 595.38 nC. As the Au sputtering time further gradually increased to 30 min, the Q_{SC} decreased continuously to 252.04 nC. **b**, When the Au sputtering time increased from 1 minute to 10 minutes, the V_{OC} of the PDC-TING decreased from 0.23 V to 0.19 V. As it further gradually increased to 30 min, the V_{OC} decreased continuously to 0.17 V.

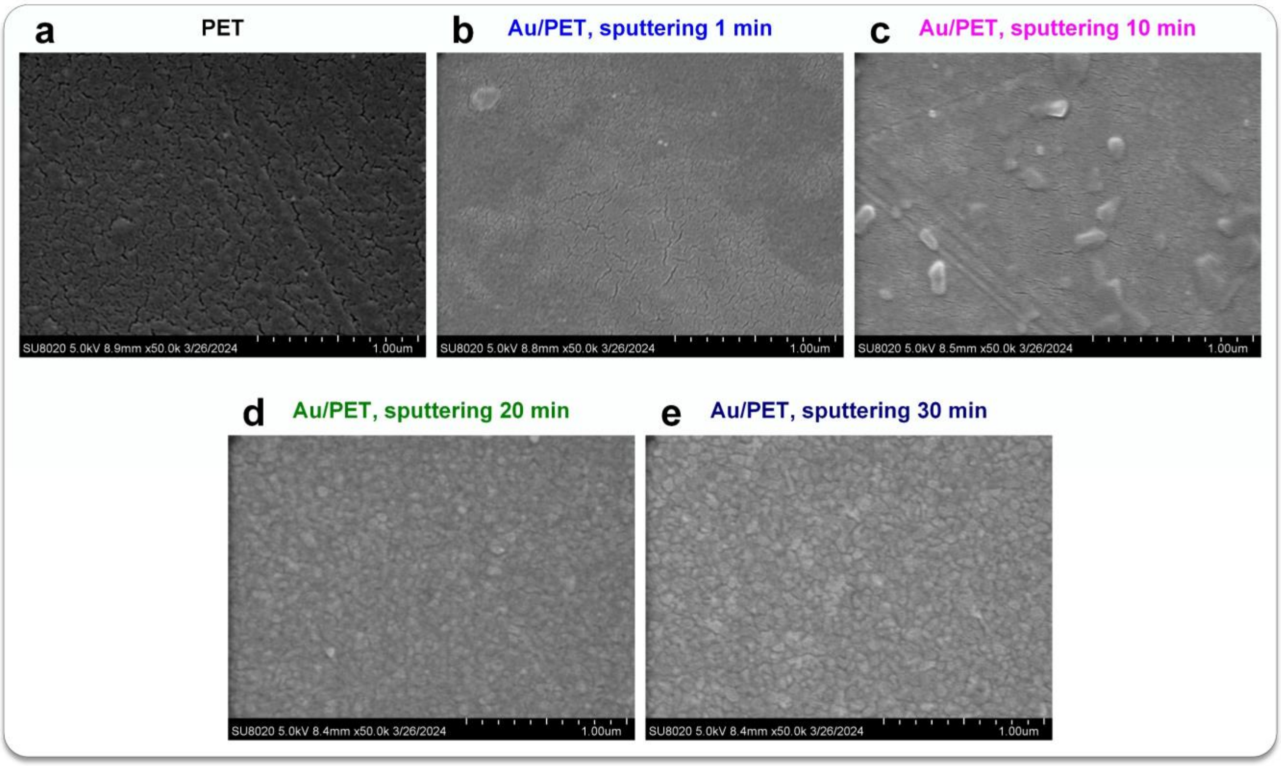

Supplementary Fig. 11 The surface morphology of the different films was observed using a scanning electron microscope (SEM). a, PET film. b Au/PET layer with the 1-minute sputtering time. **c,** Au/PET layer with the 10-minute sputtering time. **d,** Au/PET layer with the 20-minute sputtering time. **e,** Au/PET layer with the 30-minute sputtering time.

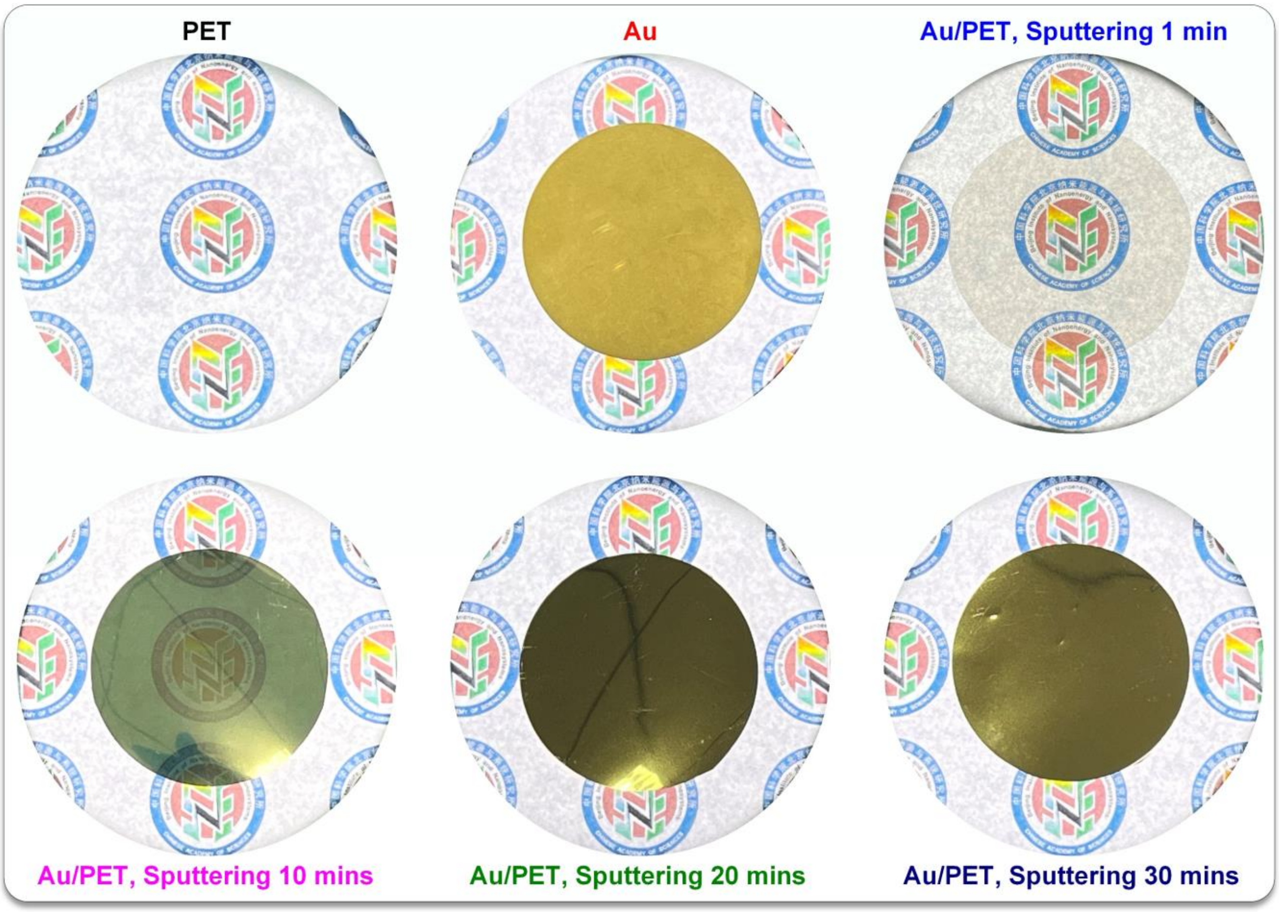

Supplementary Fig. 12 Comparison of light transmittance of the PET film, pure Au, and Au/PET layers with different sputtering times of the Au layer.

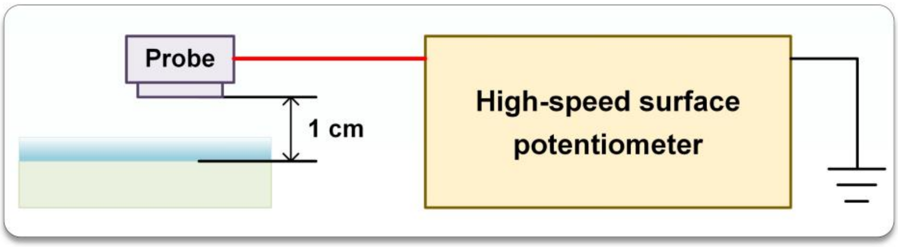

Supplementary Fig. 13 The experimental system for measuring surface potential by a high-speed surface potentiometer.

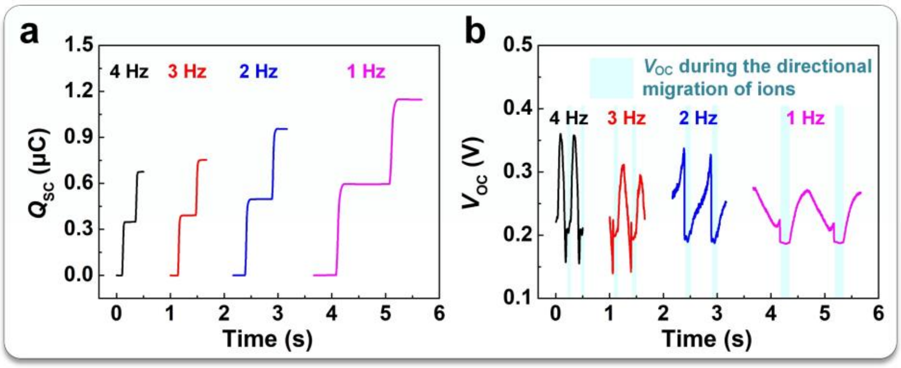

Supplementary Fig. 14 The effect of the operating frequency on the PDC-TING output was investigated. a, The comparison of the Q_{SC}. **b**, The comparison of the V_{OC}.

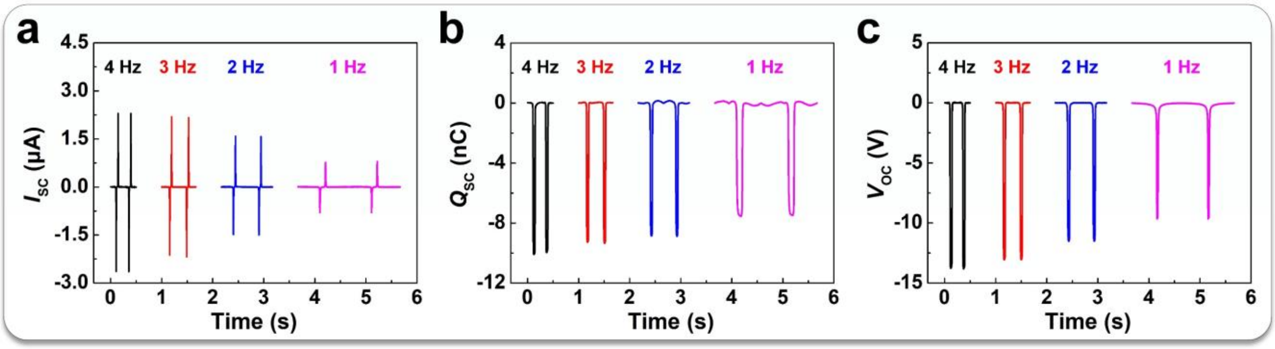

Supplementary Fig. 15 The effect of the operating frequency on the solid-solid TENG output was investigated. a, When the operating frequency of the solid-solid TENG decreased from 4 Hz to 1 Hz, the I_{SC} decreased from 2.64 μA to 0.80 μA. **b**, The Q_{SC} decreased from 10.10 nC to 7.55 nC. **c**, The V_{OC} decreased from 13.82 V to 9.66 V.

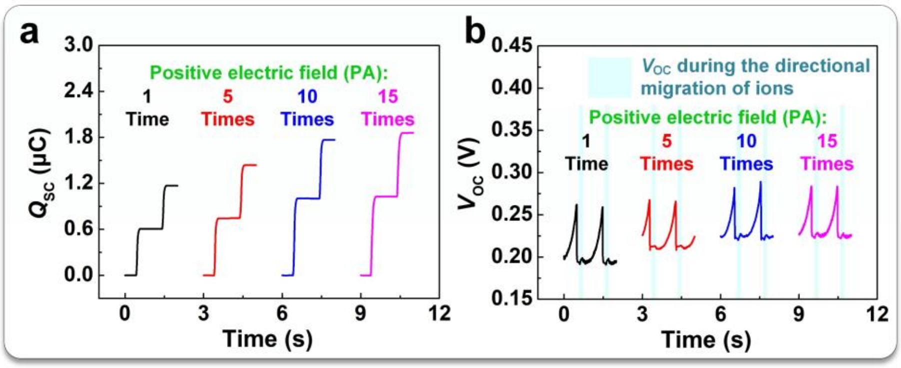

Supplementary Fig. 16 The effect of the positive electrostatic field on the PDC-TING output was investigated. a, Increasing the rubbing times from 1 to 15 times resulted in saturation of positive and negative charges on the polyamide (PA) and PTFE surface at the 10th time, respectively. Using the positive electrostatic field on the PA film could promote the PDC-TING output, increasing Q_{SC} from 0.61 μC to 1.01 μC. **b**, The V_{OC} was increased from 0.20 V to 0.23 V.

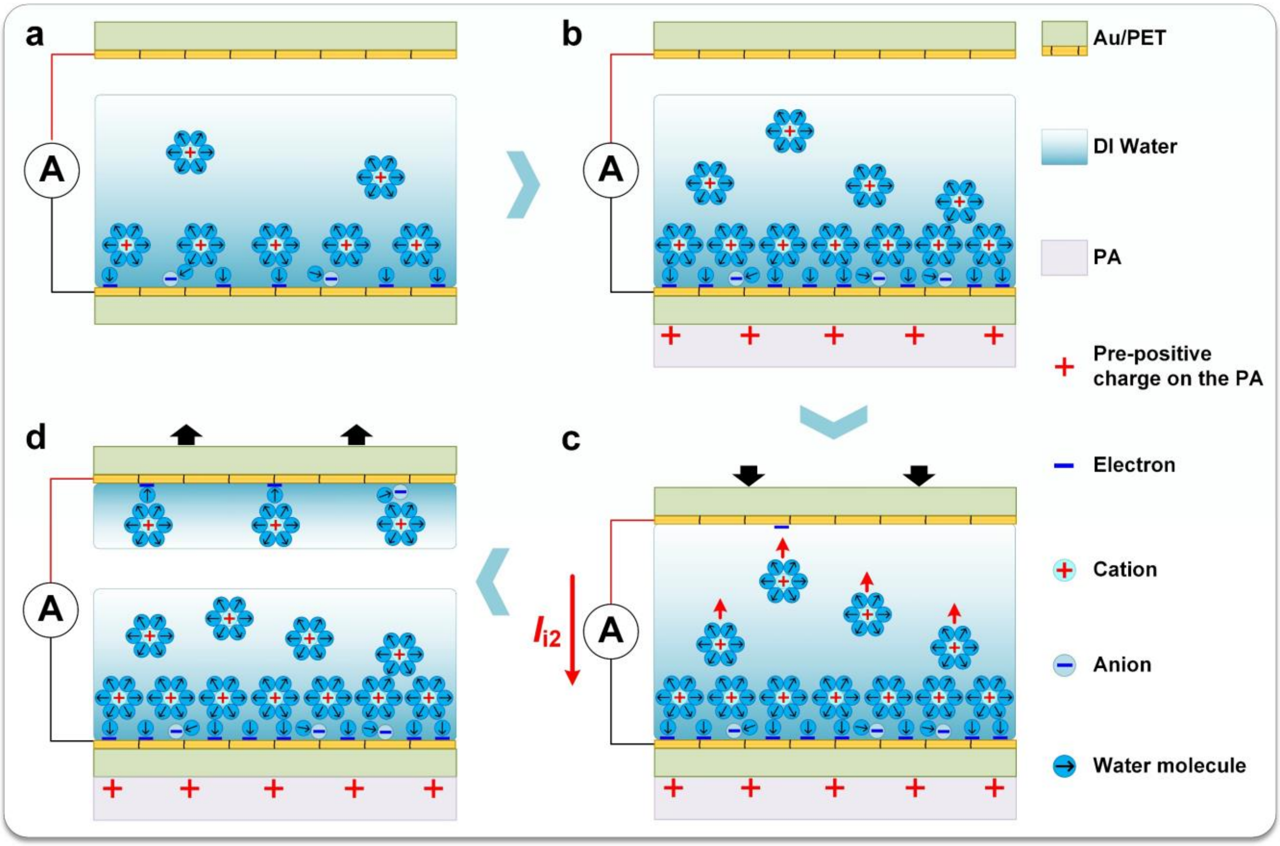

Supplementary Fig. 17 The principle of using the positive electrostatic field to adjust the PDC-TING output. a, Firstly, the DI Water fully contacted the bottom Au/PET layer, resulting in the formation of a stable and compact EDL. **b**, Secondly, under the attraction of a positive electrostatic field, the densification of the EDL was effectively increased. **c**, Thirdly, as the top Au/PET layer moved downwards to contact with water, the initial CE led to a new EDL, establishing two EDLs with significantly different symmetries. This created a higher ion concentration gradient, generating a higher ionic current I_{i2}. Fourthly, the detachment of the top Au/PET layer from the water halted ion migration. Through repeated contact and separation cycles, ion migration was consistently driven until equilibrium was reached between the two EDLs.

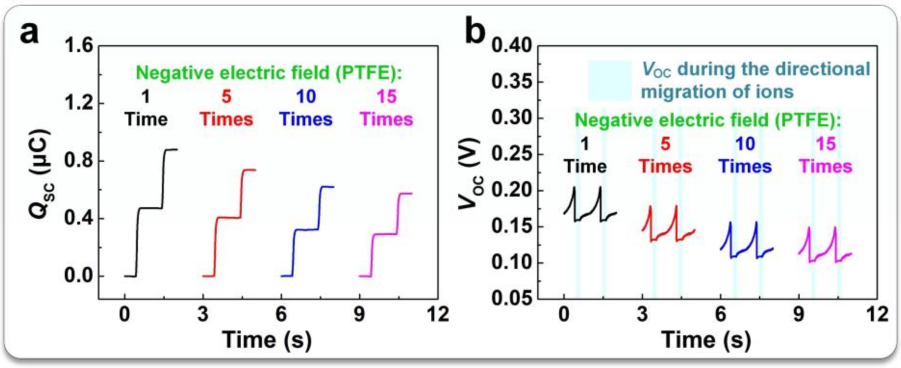

Supplementary Fig. 18 The effect of the negative electrostatic field on the PDC-TING output was investigated. a, Increasing the rubbing times from 1 to 15 times resulted in saturation of positive and negative charges on the polyamide (PA) and PTFE surface at the 10th time, respectively. Using the negative electrostatic field on the PTFE film could reduce the PDC-TING output, decreasing Q_{SC} from 0.47 μC to 0.32 μC. **b**, The V_{OC} was increased from 0.16 V to 0.10 V.

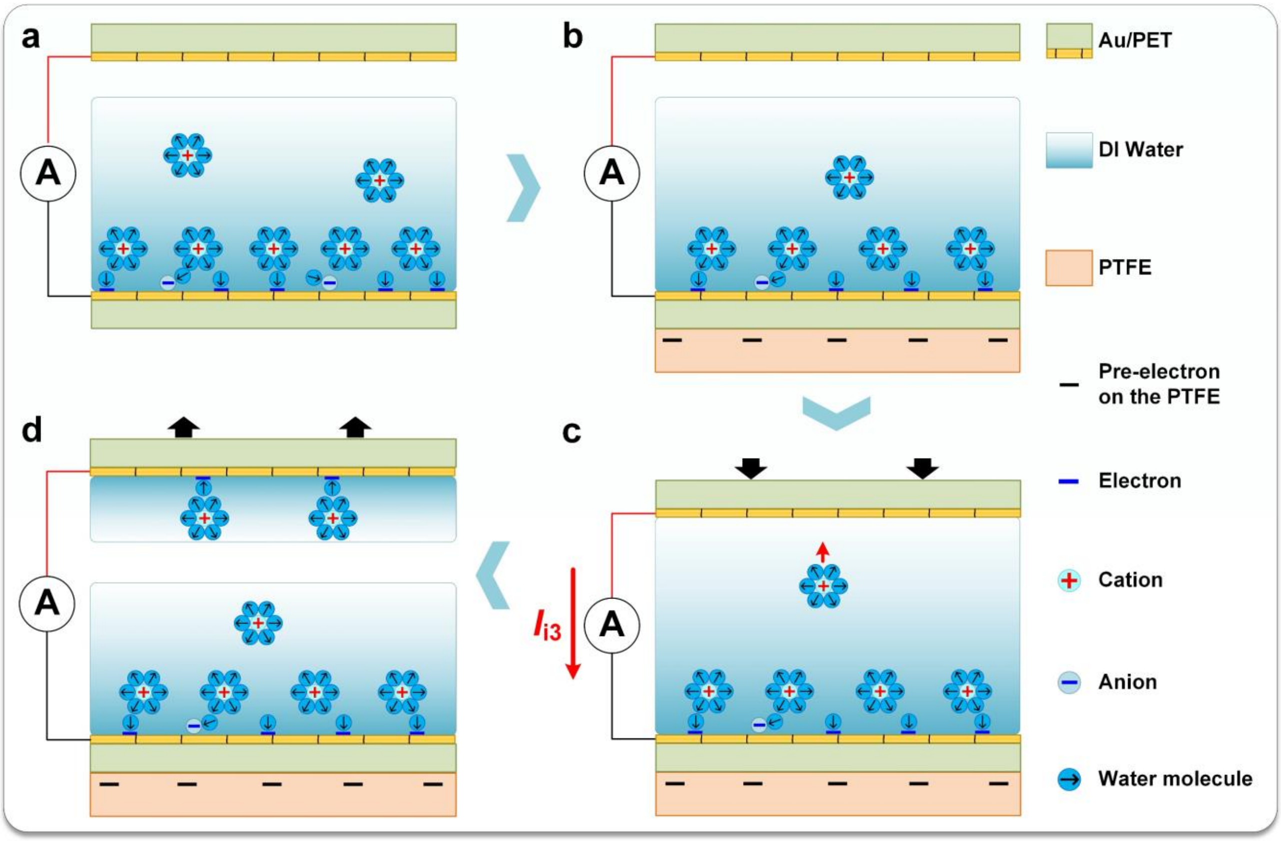

Supplementary Fig. 19 The principle of using the negative electrostatic field to adjust the PDC-TING output. a, Firstly, the DI Water fully contacted the bottom Au/PET layer, resulting in the formation of a stable and compact EDL. **b**, Secondly, under the attraction of a negative electrostatic field, the densification of the EDL was effectively decreased. **c**, Thirdly, as the top Au/PET layer moved downwards to contact with water, the initial CE led to a new EDL, establishing two EDLs with significantly different symmetries. This created a lower ion concentration, generating a lower ionic current I_{i3}. Fourthly, the detachment of the top Au/PET layer from the water halted ion migration. Through repeated contact and separation cycles, ion migration was consistently driven until equilibrium was reached between the two EDLs.

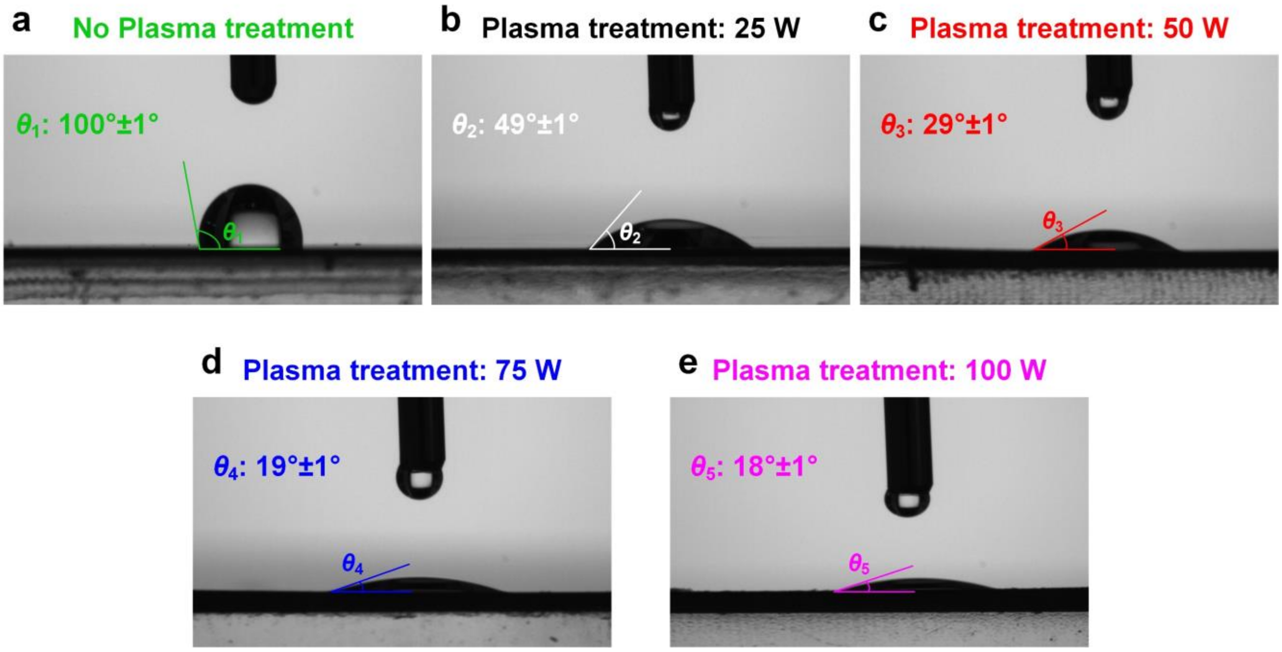

Supplementary Fig. 20 Comparison of contact angles of Au/PET layers with different Plasma treatment power. **a**, The contact angle of the initial Au/PET layer was about 100°. **b**, The contact angle of the Au/PET layer with 25-W Plasma treatment was about 49°. **c**, The contact angle of the Au/PET layer with 50-W Plasma treatment was about 29°. **d**, The contact angle of the Au/PET layer with 75-W Plasma treatment was about 19°. **e**, The contact angle of the Au/PET layer with 100-W Plasma treatment was about 18°.

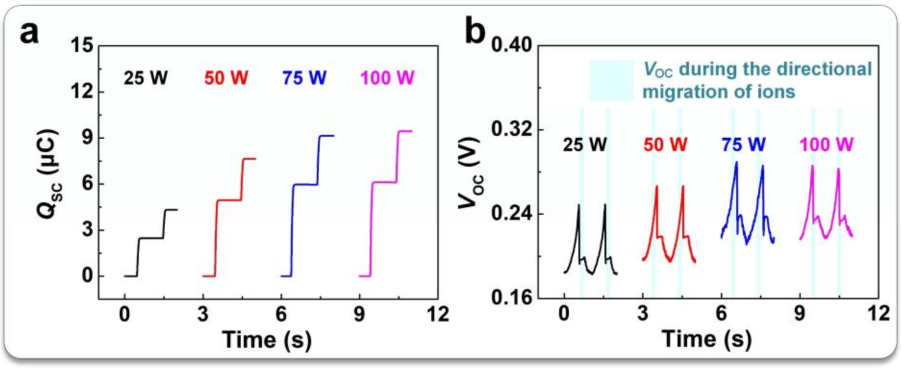

Supplementary Fig. 21 Plasma treatment of the bottom Au/PET layer was conducted to examine the impact of surface hydrophilicity on the ion concentration gradient. a, Increasing the power of Plasma treatment on the Au/PET layers from 25 W to 75 W, the Q_{SC} of the PDC-TING was increased from 2.48 μC to 5.97 μC. Beyond 75 W, further power increase did not significantly alter hydrophilicity, maintaining output stability. **b**, Increasing the power of Plasma treatment from 25 W to 75 W, the V_{OC} of the PDC-TING was increased from 0.20 V to 0.24 V.

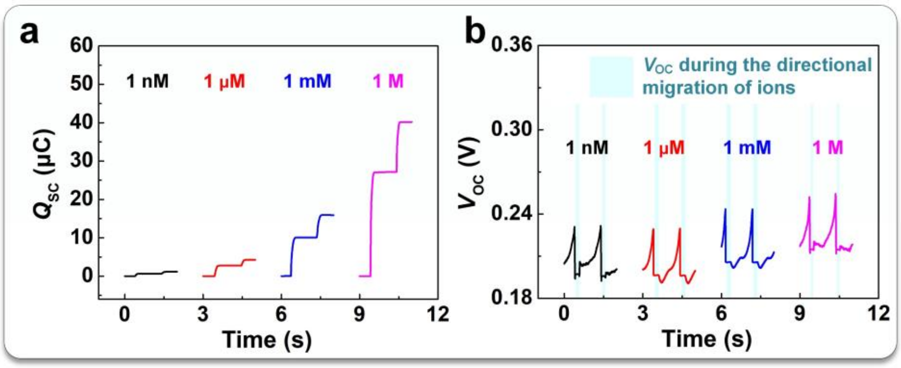

Supplementary Fig. 22 The effect of ion concentrations in the liquid on the PDC-TING output was investigated. a, When the concentration of LiCl solution was increased from 1 nM to 1 M, the Q_{SC} was increased from 0.67 μC to 27.13 μC. **b**, The V_{OC} was increased from 0.20 V to 0.22 V.

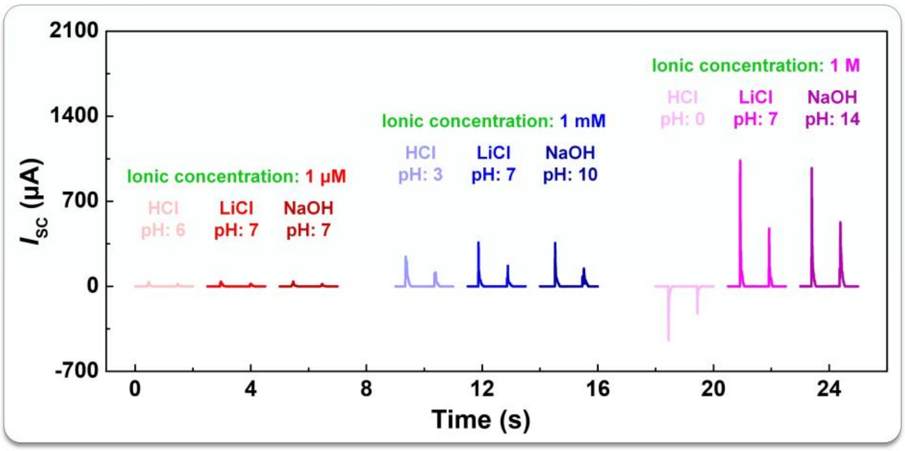

Supplementary Fig. 23 The effect of varying acidity and alkalinity of different solutions on the PDC-TING output was investigated.

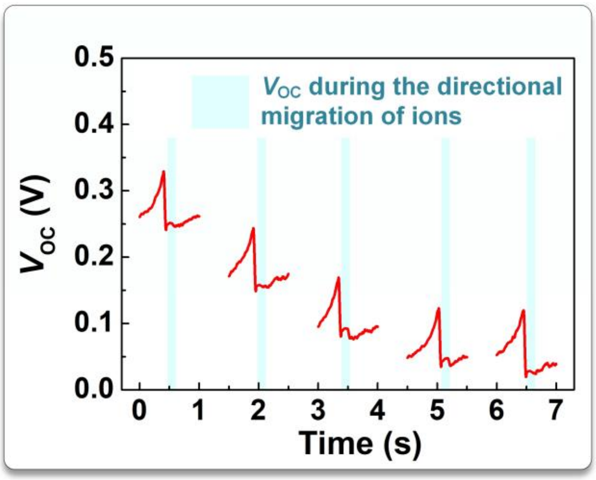

Supplementary Fig. 24 Combining the three promotion strategies of the ion concentration gradient, the V_{OC} of the PDC-TING could reach 0.25 V.

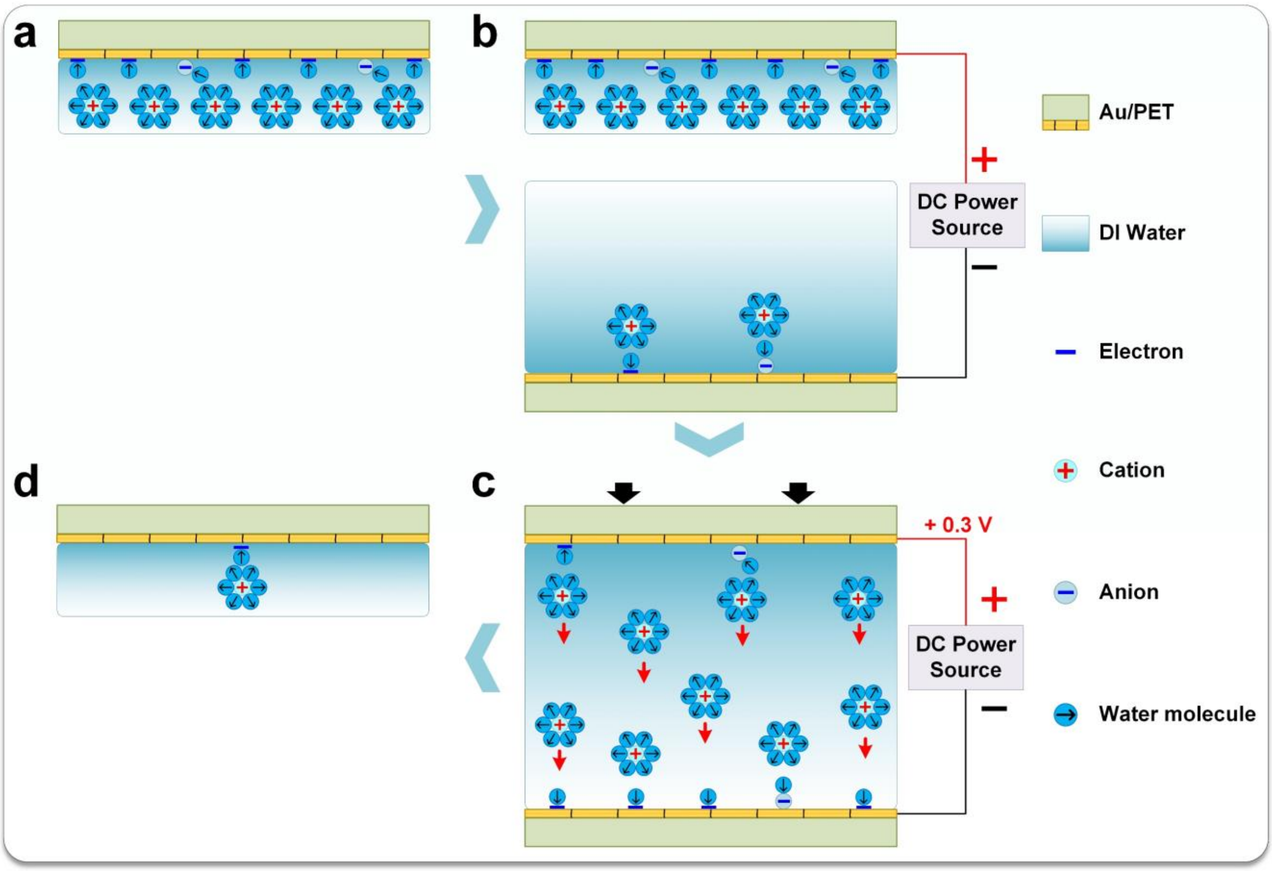

Supplementary Fig. 25 The electrochemical recovery could be used to restore the initial surface of the Au/PET layer. **a**, Firstly, the top Au/PET layer with a dense EDL was extracted from the PDC-TING. **b**, Secondly, the top Au/PET layer was placed onto a pristine bottom Au/PET layer, at which point the formation of the bottom EDL started. **c**, The forward V_{OC} of 0.3 V was applied in the external circuit between the charge-collecting layers (Au layers). The compactness of the upper EDL was gradually reduced. **d**, When the top Au/PET layer surface was recovered to the pristine state, it was extracted from the recovery system.

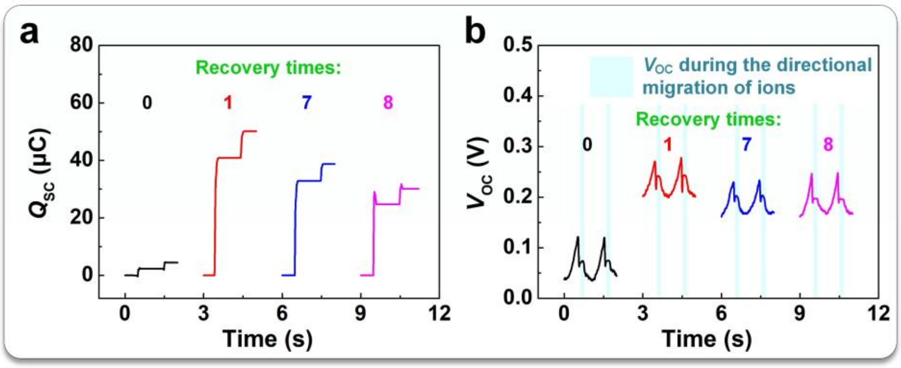

Supplementary Fig. 26 The effect of electrochemical recovery times on the PDC-TING output was investigated. a, After undergoing 7 recovery times, the PDC-TING could still generate ideal DC signals with Q_{SC} of 32.93 μC. **b**, The PDC-TING could still generate V_{OC} of 0.20 V.

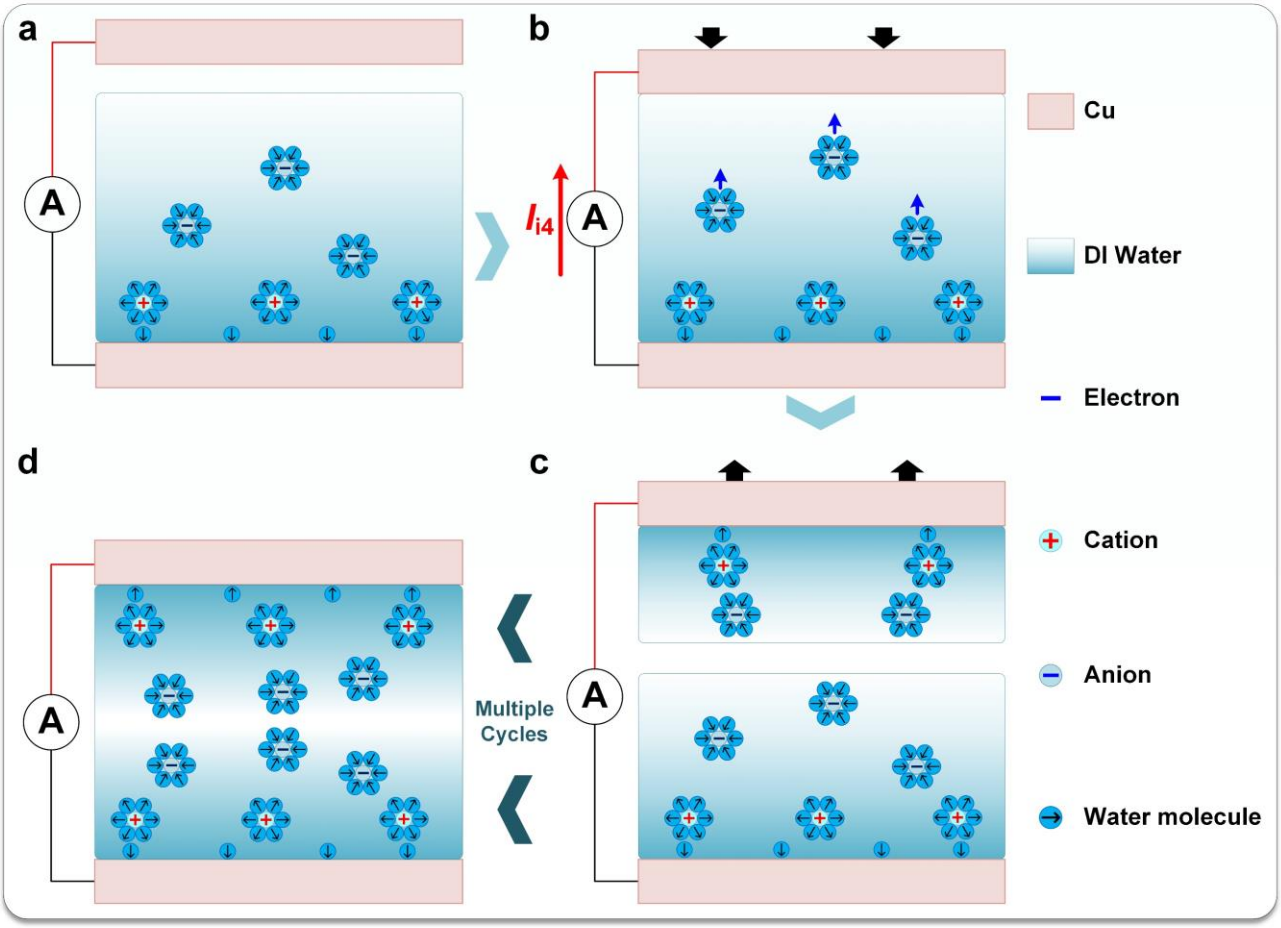

Supplementary Fig. 27 Through temporally controlling EDL formation at the contact interface between DI Water and pure metal, it could construct the ion concentration gradient to induce directional migration of anions, thereby enabling metal-based PDC-TING. a, Firstly, when DI water contacted the bottom pure metal (Copper, Cu) layer surface, a stable EDL was formed. **b**, Secondly, as the top Cu layer moved downwards to contact with water, the initial CE led to a new EDL, establishing two EDLs with significantly different symmetries. This created an ion concentration gradient driving anions in water to migrate directionally upwards, facilitating electron transfer in the external circuit and generating an ionic current I_{i4}. **c**. Thirdly, the detachment of the top Cu layer from the water halted ion migration. **d**, Through repeated contact and separation cycles, anion migration was consistently driven until equilibrium was reached between the two EDLs.

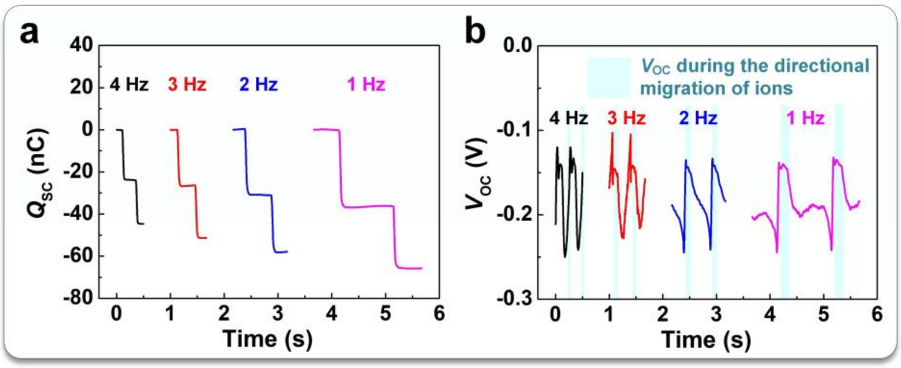

Supplementary Fig. 28 The effect of operating frequency on the metal-based PDC-TING output was investigated. a, The comparison of the Q_{SC}. **b**, The comparison of the V_{OC}.

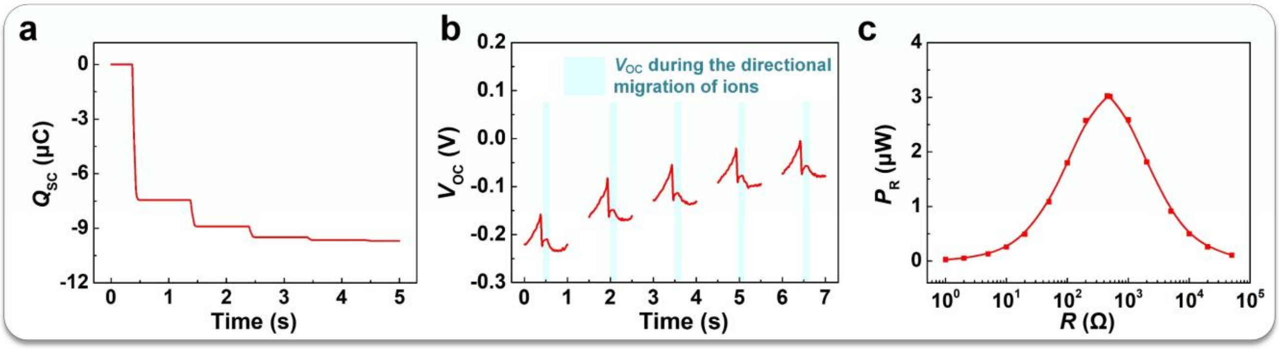

Supplementary Fig. 29 Combining the three promotion strategies of the ion concentration gradient, the optimal negative output performance of metal-based PDC-TING was tested. a, The Q_{SC} could reach -7.45 μC. **b**, The V_{OC} could reach -0.21 V. **c**, The peak power (P_R) could reach 3.03 μW.

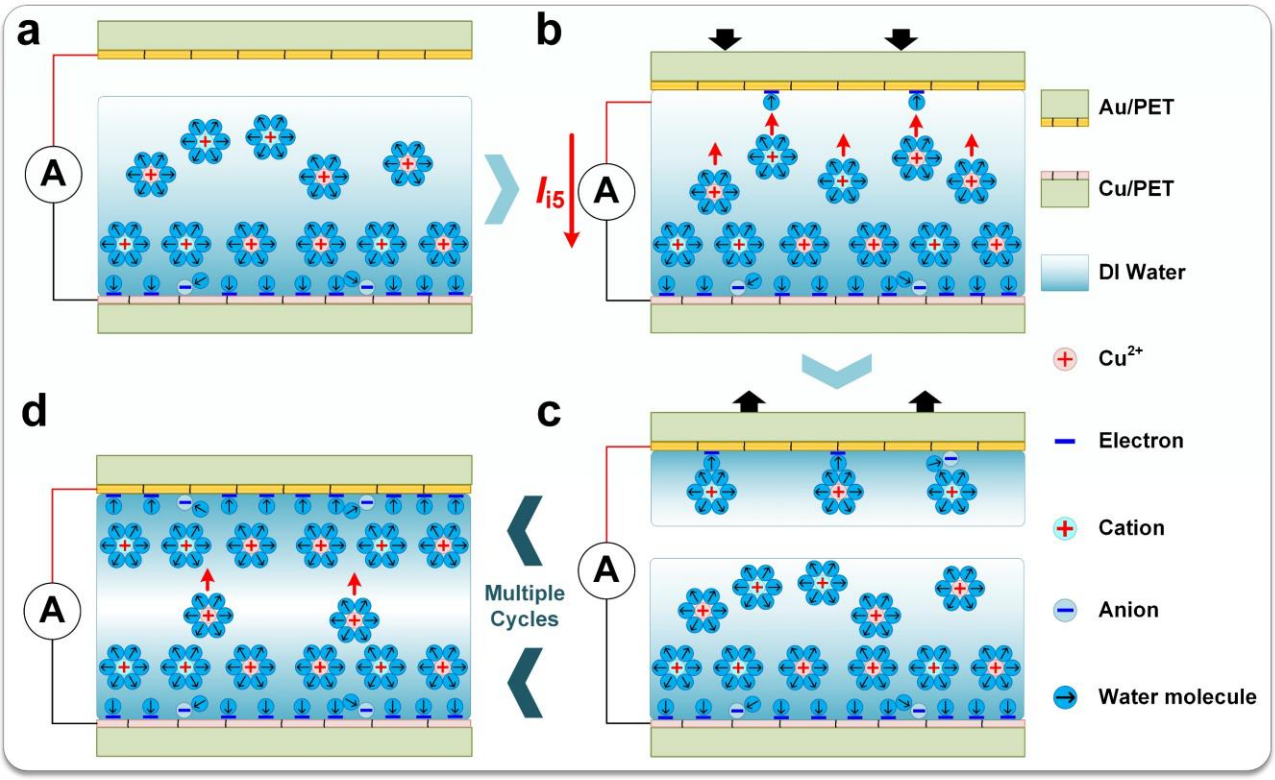

Supplementary Fig. 30 The operation principle of the SDC-TENG. a, Firstly, the DI water fully contacted the bottom Cu/PET layer, resulting in the formation of a denser EDL. **b**, Secondly, as the top Au/PET layer moved downwards to contact with water, the initial CE led to a new EDL, establishing two EDLs with significantly different symmetries. The ion concentration gradient formed by temporal controlling EDL formation and the chemical potential difference generated by the redox reaction could produce a synergistic effect, thereby jointly promoting the rapid directional migration of ions and generating an efficient ionic current I_{i5}. **c**, Thirdly, the detachment of the top Au/PET layer from the water halted ion migration **d**, After multiple contact and separation cycles, the ion concentration gradient formed by temporal controlling EDL formation disappeared, and the chemical potential difference generated by the redox reaction could continue to drive the directional migration of ions, resulting in a lower stable current output.

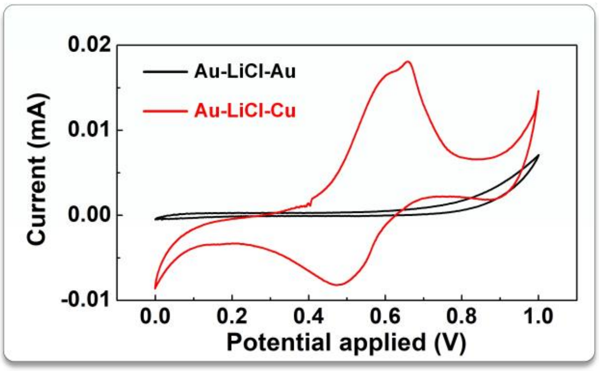

Supplementary Fig. 31 In 1 M LiCl solution, the cyclic voltammetry curves between the two Au/PET layers and between the Au/PET layer and Cu/PET layer were compared.

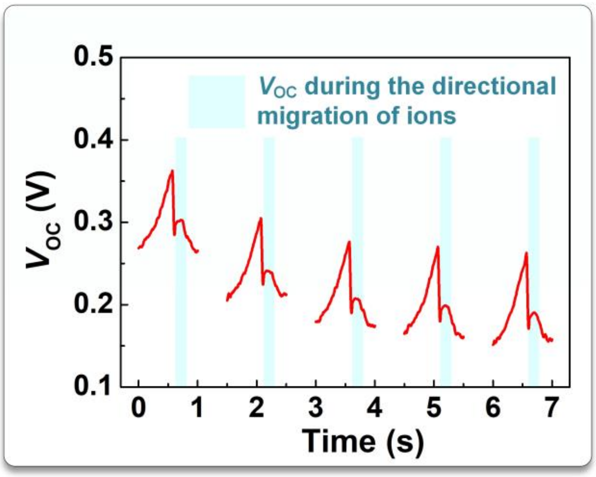

Supplementary Fig. 32 The V_{OC} of the SDC-TING could reach 0.30 V.

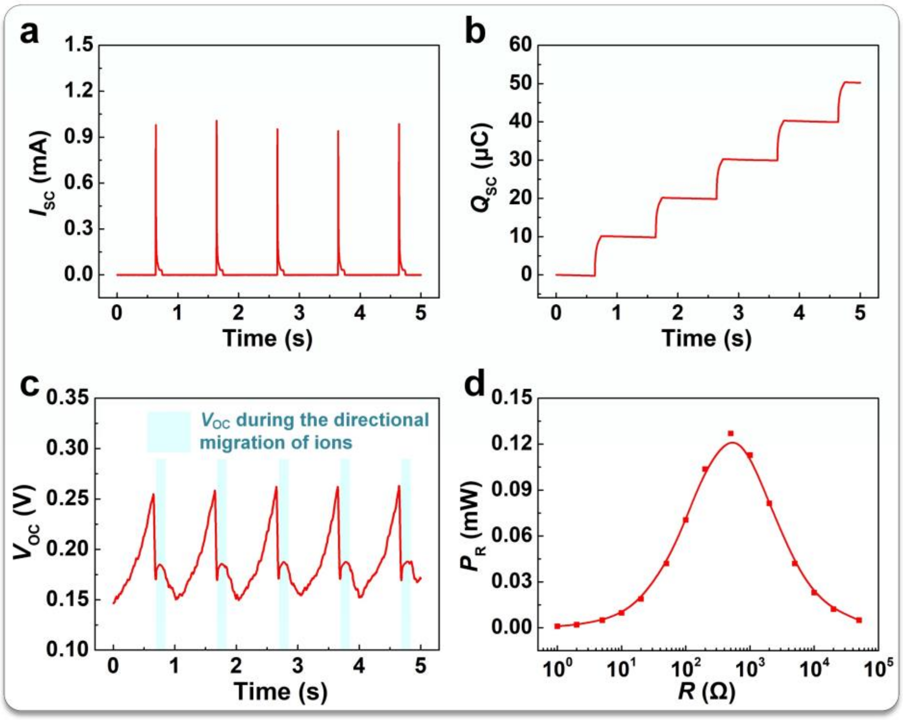

Supplementary Fig. 33 After the disappearance of the ion concentration gradient caused by temporally controlling EDL formation, the PDC-TING output depended solely on the redox reaction. **a**, The I_{SC} was 1.01 mA. **b**, The Q_{SC} was 10.10 μC. **c**, The V_{OC} was 0.18 V. **d**, The P_R was 0.13 mW.

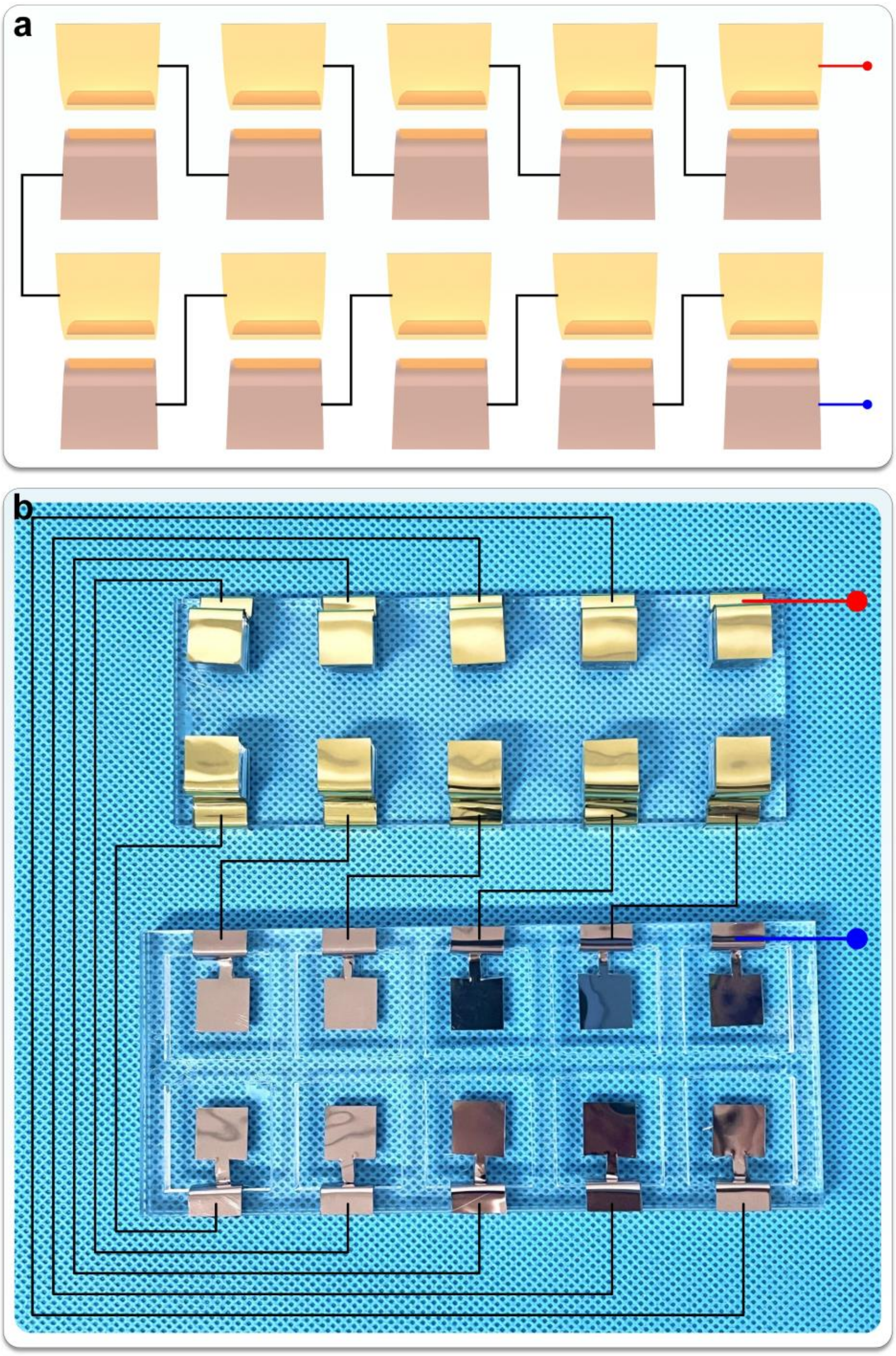

Supplementary Fig. 34 The 10-node connecting in series of the SDC-TINGs was displayed. a, The schematic diagram of the 10-node connecting in series of the SDC-TINGs. **b**, The physical diagram of the 10-node connecting in series of the SDC-TINGs.

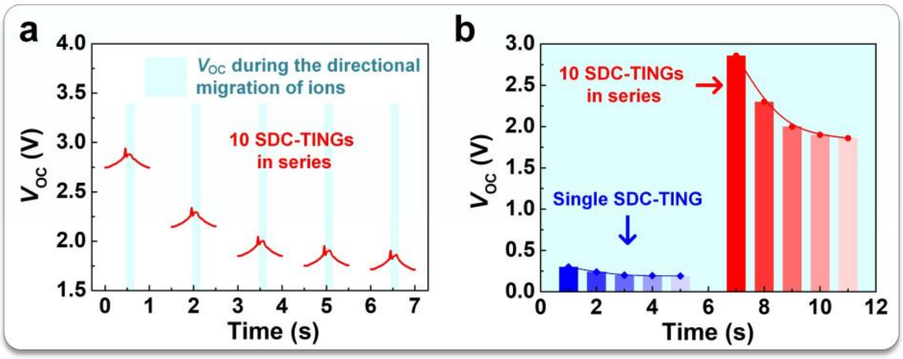

Supplementary Fig. 35 The V_{OC} of SDC-TING could be effectively improved by the 10-node connecting in series. a, The SDC-TINGs in series could generate a V_{OC} of 2.88 V. **b**, The V_{OC} generated by a single SDC-TING and SDC-TINGs in series was compared.

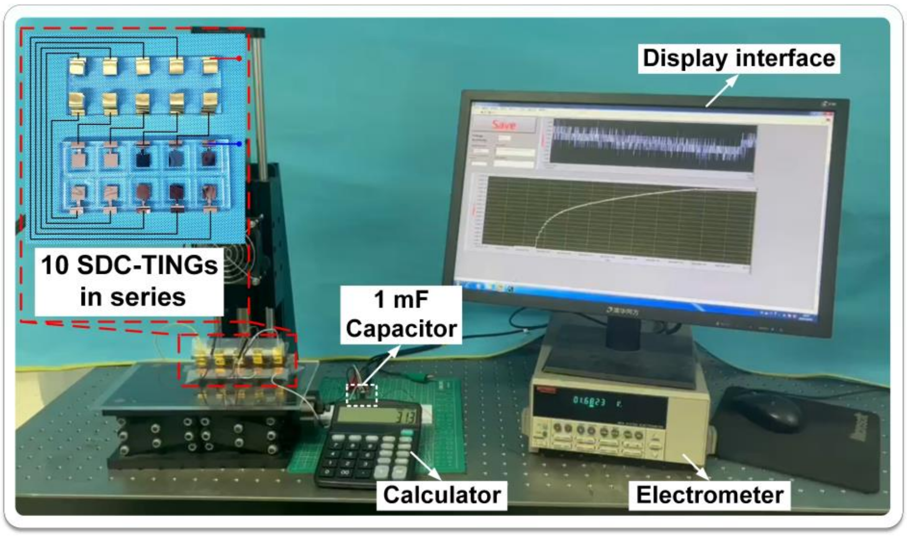

Supplementary Fig. 36 The pristine SDC-TINGs in series could charge the 1 mF capacitor to 1.5V within 60 seconds and ensure that the calculator was operating normally.

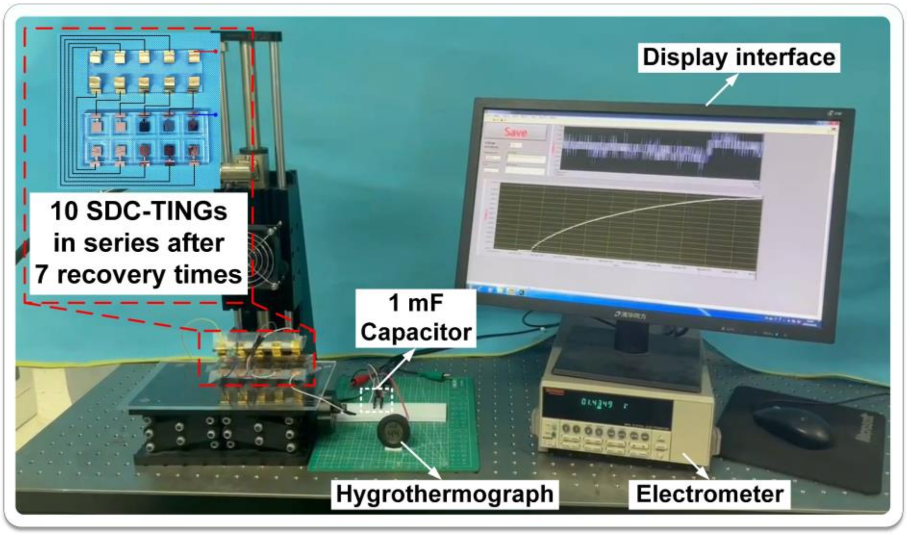

Supplementary Fig. 37 The SDC-TINGs in series after 7 recovery times could still charge it to 1.4V and ensure that the hygrothermograph was operating normally.

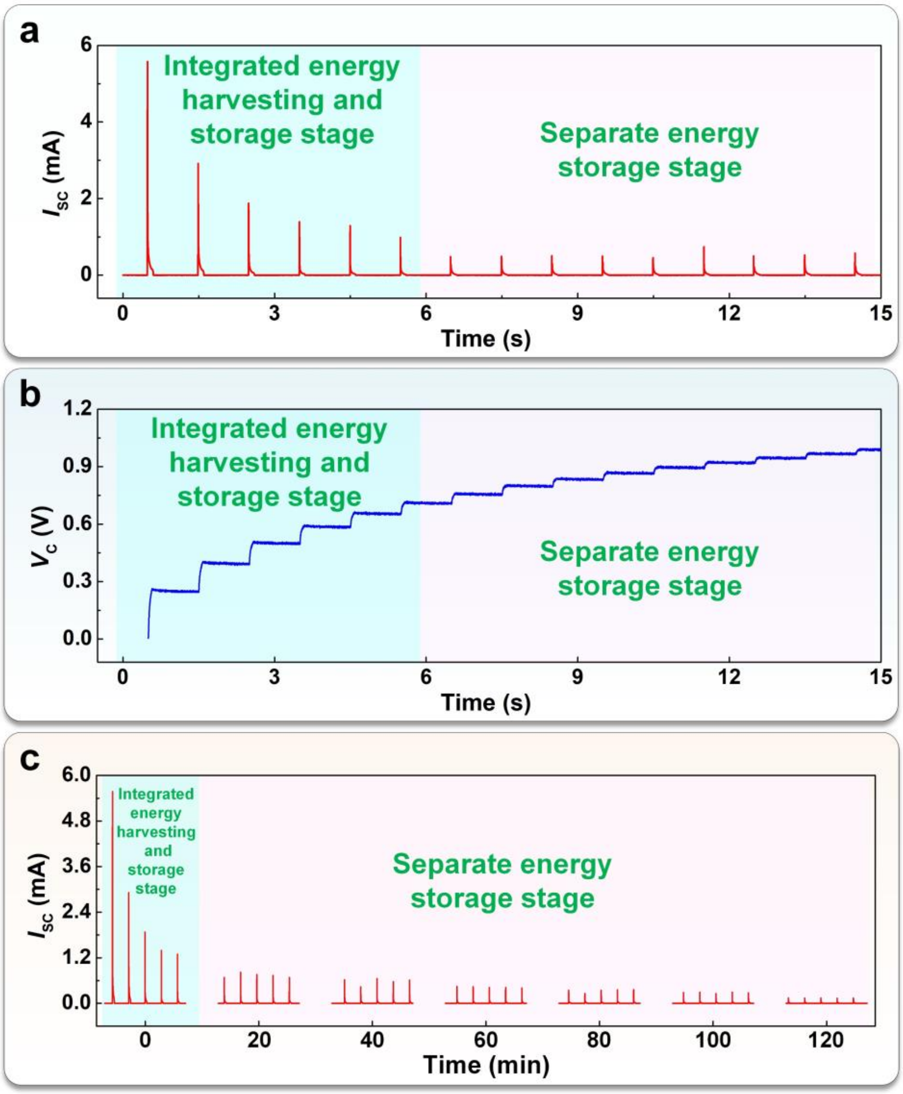

Supplementary Fig. 38 In the field of energy, the SDC-TING could operate in two interrelated stages: the integrated energy harvesting and storage stage, followed by the separate energy storage stage. **a**, The I_{SC} generated by the SDC-TING in two interrelated stages was compared. **b**, The charging performance of the SDC-TING for 1 mF capacitor in two interrelated stages was compared. **c**, The subsequent separate energy storage stage substantially enhanced the stability of the SDC-TING.
