# Supplementary material for: Triboiontronics with temporal control of electrical double layer formation
Source: Nat Commun. 2024 Jul 23;15:6182. doi: 10.1038/s41467-024-50518-3 (PMC11263338; doi:10.1038/s41467-024-50518-3)
Supplement: Supplementary file 3 — Description of Additional Supplementary Files [file 41467_2024_50518_MOESM3_ESM.pdf]

Description of Additional Supplementary Files

File Name: Supplementary Movie 1
Description: Ten SDC-TINGs in series ensured the stable operation of the calculator

File Name: Supplementary Movie 2
Description: Ten SDC-TINGs in series after 7 recovery times ensured the stable operation of the hygrothermograph
